# Adaptive governance for climate-resilient urban health systems: qualitative insights from stakeholders in Dar es Salaam, Tanzania

**DOI:** 10.1016/j.dialog.2026.100359

**Published:** 2026-10-05

**Authors:** Lawrencia D. Mushi, Noel O. Ogweyo

**Affiliations:** Department of Health Systems Management, School of Public Administration and Management, Mzumbe University, Morogoro, Tanzania

**Keywords:** Adaptive governance, Climate change adaptation, Urban health systems, Climate resilience, Tanzania

## Abstract

**Background:**

Climate change poses growing risks to urban health systems, particularly in rapidly growing cities in low- and middle-income countries. Although strengthening climate resilience has become a global priority, evidence remains limited on how governance processes support adaptation within urban health systems. This study explored stakeholder perspectives on adaptive governance for climate change within Dar es Salaam's urban health system in Tanzania.

**Methods:**

An exploratory qualitative study was conducted in Dar es Salaam, Tanzania, between October and November 2025. Data were collected through 38 in-depth interviews and five focus group discussions involving participants from municipal health management teams, public health facilities, and academia. Participants were purposively selected based on their roles in health governance, service delivery, climate change adaptation, and research. Interviews and discussions were conducted in Kiswahili, audio-recorded, transcribed verbatim, translated into English, and analysed thematically using ATLAS.ti software. The Adaptive Governance Framework guided the interpretation of the findings.

**Findings:**

Three interrelated themes emerged from the analysis. *First*, multi-sectoral networks and coordination: Stakeholders perceived adaptation governance as operating through dynamic multi-sectoral networks, where coordination was understood as a relational process involving negotiation, trust-building, and information exchange across institutions and actors. *Second*, Institutional perspectives on community engagement and knowledge integration: Participants viewed community engagement as an important governance interface that links institutional responses to local knowledge, risk awareness, and adaptation actions. *Third*, leadership, institutional learning and responsiveness in climate adaptation governance: Leadership was perceived as a bridging mechanism that facilitates learning, mediates between policy intention and practice, and enables institutional responsiveness through continuous adaptation rather than through formal authority alone.

**Interpretation:**

The findings highlight adaptive governance within urban health systems as a process of relational coordination, knowledge integration, and learning-oriented leadership, rather than solely the presence of institutional structures or resources. Strengthening climate resilience requires governance arrangements that promote collaborative relationships, enable knowledge exchange between communities and institutions, and support leadership practices that promote learning and adaptive responses. These insights extend current understanding of adaptive governance by revealing how stakeholders perceive and experience governance processes in the context of urban health systems in sub-Saharan Africa. However, the findings reflect the perspectives of institutional stakeholders in a single urban context and do not include direct perspectives from community members.

## 1 Introduction

Climate change is increasingly recognized as one of the greatest threats to global health and sustainable development in the twenty-first century [1], [2]. Rising temperatures, extreme weather events, flooding, droughts, and changing patterns of climate-sensitive diseases are placing growing pressure on health systems worldwide, specifically in low- and middle-income countries (LMICs) where resources are limited [3], [4], [5], [6]. According to a projection in the 2024 World Health Economic Forum report, climate change could result in 14.5 million deaths and $12.5 trillion in economic losses, disproportionately affecting LMICs.(2) Urban areas are especially vulnerable because rapid population growth, unplanned settlements, inadequate infrastructure, and environmental degradation interact with climate hazards to amplify health risks [7], [8], [9], [10]. In sub-Saharan Africa (SSA), cities are experiencing recurrent climate-related health challenges, including outbreaks of waterborne and vector-borne diseases, heat-related illnesses, injuries, and disruptions to essential health services [7], [9], [11], [12]. These challenges require health systems together with other climate-determining sectors not only to respond to immediate health threats but also to adapt their governance arrangements to manage increasingly complex and uncertain risks.

Building climate-resilient health systems requires more than technical interventions, infrastructure investments, or disease surveillance systems [2], [13], [14]. Effective adaptation depends on governance processes that enable coordination across sectors, participation of diverse stakeholders, integration of knowledge, and institutional learning [2], [7], [13], [15]. Climate-related health risks emerge through interactions among environmental, social, economic, and political systems, making them difficult for any single institution or sector to address independently [15], [16]. Consequently, there is growing recognition that adaptive governance characterized by collaboration, flexibility, shared decision-making, and continuous learning is essential for strengthening resilience to climate change [15], [16], [17]. Chaffin et al. describe adaptive governance as interactions among actors, networks, organizations, and institutions that enable collective responses to changing conditions [18], while subsequent scholarship has emphasized that adaptation is also shaped by power, competing interests, and struggles over whose knowledge and priorities influence decision-making [19], [20]. Adaptive governance frameworks therefore emphasize the importance of linking actors and institutions across multiple levels of governance, which require collective action, and creating mechanisms that enable systems to respond and adjust to changing conditions [2], [13], [16], [17].

Despite increasing attention to climate change adaptation, evidence on how adaptive governance is understood and practised, particularly within urban health systems across LMICs, remains limited. Existing studies have primarily examined climate vulnerability, health system preparedness, adaptation planning, adaptation approaches, governance arrangements, and barriers to implementing climate change adaptation within health systems [2], [7], [15], [16], [21], [22], [23], [24], [25], [26], [27], [28]. However, comparatively little attention has been given to how stakeholders perceive and experience the governance processes that shape climate change adaptation within urban health systems. Understanding how different actors perceive governance arrangements, collaboration mechanisms, community participation, and institutional responsiveness is important for identifying opportunities to strengthen adaptive capacity within health systems. This knowledge gap is important, especially in rapidly growing African cities, where climate risks are intensifying, and governance systems must operate across multiple sectors, institutions, and administrative levels.

Moreover, recent climate and health scholarship from the Global South further emphasizes the need for locally generated evidence to inform climate adaptation and health policy, given substantial differences in vulnerabilities, institutional contexts, and policy environments across regions [29], [30], [31]. For example, evidence from Latin America highlights the importance of intersectoral collaboration and health-centred climate-resilient development in addressing climate risks and social inequities, while research on climate adaptation policies emphasizes the need for context-specific evidence to guide national and subnational responses [29], [30], [31]. These gaps emphasize the importance of generating empirical evidence from rapidly growing African cities, where climate risks intersect with distinctive institutional, infrastructural, and social conditions [7].

While recent research has examined institutional capacity gaps within urban health systems in Dar es Salaam, including weaknesses in early warning systems, human resources, and infrastructure [7], [22], the governance processes through which adaptation is perceived and enacted remain underexplored. Adaptive governance theory suggests that resilience depends not only on the availability of resources and structures but also on the quality of relationships, learning processes, and collaborative arrangements that enable systems to respond to change [16], [17]. Importantly, scholarship on adaptation also highlights that these processes are shaped by power, institutional interests, and whose knowledge and priorities are recognized in decision-making [19], [20]. However, empirical evidence on how these governance processes operate within urban health systems in sub-Saharan Africa is scarce.

Dar es Salaam, Tanzania's largest city and economic hub, is increasingly exposed to climate-related hazards including flooding, heat stress, environmental degradation, and outbreaks of climate-sensitive diseases [7], [22], [25], [32], [33]. These risks are intensified by rapid urban growth, the expansion of informal settlements that accommodate approximately 70% of the city's population, persistent socioeconomic vulnerabilities, and inadequate infrastructure, including poorly developed stormwater drainage systems [32]. These challenges require coordinated responses involving health authorities, local government institutions, communities, academic institutions, and other sectors beyond health [17]. Guided by the Adaptive Governance Framework [17], the primary objective of this study was to explore how stakeholders perceive and experience governance processes that shape climate change adaptation within Dar es Salaam's urban health system. Specifically, the study examined stakeholders' perspectives on (i) multi-sectoral coordination and networks, (ii) community participation and knowledge integration, (iii) leadership and institutional learning, and (iv) institutional responsiveness in climate adaptation governance.

This study forms part of a broader qualitative research programme examining governance of climate change adaptation in urban Dar es Salaam. An earlier analysis of the same dataset examined health system capacity gaps for climate change adaptation governance and their implications for resilience [7]. The present analysis addresses a complementary but distinct question by examining how stakeholders perceive and experience the governance processes through which adaptation is coordinated and enacted, with particular attention to multi-sectoral networks, community participation, leadership, learning, knowledge exchange, and institutional responsiveness.

## 2 Methods

### 2.1 Study design

This study employed an exploratory qualitative design to explore stakeholder perspectives on adaptive governance for climate change adaptation within the urban health system of Dar es Salaam, Tanzania. A qualitative exploratory design was considered appropriate because it enabled an in-depth exploration of stakeholders' perspectives and experiences regarding climate change adaptation governance within the urban health system. This approach was specifically suitable given the limited empirical evidence on adaptive governance in urban health systems in the LMICs [34]. The study was guided by the Adaptive Governance Framework proposed by Folke and colleagues, which emphasizes multi-actor collaboration, learning, participation, leadership, and institutional flexibility in responding to complex and uncertain environmental challenges [17]. The study was reported in accordance with the Consolidated Criteria for Reporting Qualitative Research (COREQ) checklist [35]. (*Supplemental file _Research checklist*).

### 2.2 Study setting and participants

The present article is a secondary qualitative analysis of data generated from a larger qualitative study on governance of climate change adaptation in urban Dar es Salaam. The same dataset was used in our previously published article which examined health system capacity gaps for climate change adaptation governance, focusing on preparedness mechanisms, human resources, infrastructure, financing, surveillance, and operational readiness [7]. The present analysis addresses a distinct research question and analytical focus examining how stakeholders perceive and experience adaptive governance processes. The study was conducted in Dar es Salaam, Tanzania's largest city and commercial centre, which is characterized by rapid urbanization and increasing exposure to climate-related health risks. The city experiences recurrent flooding, rising temperatures, environmental degradation, and outbreaks of climate-sensitive diseases such as malaria, cholera, and respiratory infections [7], [22], [25], [32], [33]. These challenges are compounded by high population density, expanding informal settlements, and growing demand for health services [7], [22], [25], [32], [33], making Dar es Salaam an appropriate setting for examining governance processes related to climate adaptation within urban health systems.

Participants were purposively selected based on their roles, responsibilities, and experience in health governance, climate change adaptation, policy implementation, health service delivery, or climate-health training and research. The study included healthcare providers from public health facilities, members of municipal health management teams, and university lecturers involved in climate change, public health, and health systems research. Participants were recruited from one public health university, four hospitals, six health centres, and eight dispensaries located across four municipal councils in Dar es Salaam. Potential participants were identified based on the predefined eligibility criteria related to health systems governance and service delivery for climate related disease and then approached through consultation with their respective institutions and leadership. Participation was voluntary, and all individuals who were approached and eligible agreed to participate; no participant declined or withdrew from the study.

A total of 68 participants were included in the study. 38 participants took part in in-depth interviews (IDIs), while 30 participated in 5 focus group discussions (FGDs). IDIs included participants with individual responsibilities and experiences relevant to health governance, climate change adaptation, service delivery, and research or policy, whereas FGDs were conducted with frontline healthcare providers to capture shared experiences and perspectives within health facility settings. Recruitment continued until thematic saturation was achieved, defined as the point at which no substantially new concepts, themes, or insights emerged from successive interviews and discussions. Saturation was assessed iteratively during data collection through ongoing review of emerging codes, categories, and themes. After successive interviews and FGDs, the research team assessed whether new data were generating substantially new concepts relevant to the study objectives. As data collection progressed, subsequent interviews and FGDs confirmed existing patterns rather than generating new significant concepts, and the research team therefore judged that thematic saturation had been reached.

### 2.3 Data collection

Data were collected between October and November 2025 using IDIs and FGDs. Data were collected using interview and discussion guides developed by all authors. The data collection guides were developed for the larger study on governance of climate change actions in urban health systems in Dar es Salaam. The guides were informed by the study objectives and key concepts of the Adaptive Governance Framework, including stakeholder participation, coordination, learning, leadership, institutional responsiveness, and multi-sectoral collaboration. Three semi-structured guides were used: an FGD Guide, an IDI Guide for health system stakeholders (including healthcare workers and Council/Municipal Health Management Teams), and an IDI Guide for university stakeholders. The full guides are provided as supplementary materials (*Supplementary File 1: FGD Guide; Supplementary File 2: IDI Guide 1 – Health System Stakeholders; and Supplementary File 3: IDI Guide 2 – University Stakeholders*).

Across the three guides, the main question domains covered: (i) experiences and perceptions of climate-related health risks and their effects on health services; (ii) preparedness and response to climate-related health events; (iii) awareness, interpretation, and implementation of relevant policies and procedures; (iv) institutional roles, stakeholder engagement, collaboration, and coordination; (v) community-based solutions and local knowledge; (vi) leadership, institutional capacity, and challenges to implementation; and (vii) recommendations for strengthening governance of climate change adaptation in urban health systems. Open-ended questions and probes were used to explore participants' experiences, perceptions, examples, challenges, and suggestions in greater depth. The guides were pre-tested among health professionals outside the study sites and refined to improve clarity, sequency and relevance.

A total of 38 IDIs were conducted, including 36 face-to-face interviews at participants' workplaces and two virtual interviews conducted at participants' convenience. Interviews lasted between 28 and 59 min. Five FGDs were conducted with frontline healthcare providers to explore shared experiences and collective perspectives on climate adaptation governance within health facilities and communities. The FGDs were conducted in private meeting spaces within health facilities and lasted between 57 and 86 min. All interviews and discussions were conducted in Kiswahili, audio-recorded with participants' permission, and supplemented with detailed field notes documenting non-verbal cues, contextual observations, and emerging reflections. All data were collected by the first author, a female senior lecturer, with assistance from the second author, a male tutorial assistant at the university.

### 2.4 Data management and analysis

Audio recordings from the interviews and focus group discussions were transcribed verbatim by the second author within 24 h of data collection. Transcripts were reviewed against the original recordings by the interviewer and note-taker to ensure completeness and accuracy. The Kiswahili transcripts were subsequently translated into English and cross-checked against the original recordings to preserve meaning and contextual realities. All data were anonymized and stored on password-protected computers accessible only to the research team.

Data were analysed thematically using ATLAS.ti software. The analysis followed the six-phase approach proposed by Braun and Clarke [36], as also applied in previous analyses [7]. Coding was primarily inductive. Although the data collection guides were informed by key concepts from the Adaptive Governance Framework, these concepts were not used as predetermined coding categories. Instead, codes and themes were developed from participants' accounts, allowing unexpected concepts and patterns to emerge from the data. The Adaptive Governance Framework was subsequently used as an interpretive lens for relating the emergent findings to concepts of networks, participation, learning, leadership, and institutional responsiveness.

First, transcripts were read repeatedly to facilitate familiarization with the data. Data familiarization began during data collection where the authors reviewed the audio recordings to determine the emerging concepts and data saturation. Second, initial codes were generated inductively by identifying meaningful textual units related to governance processes, stakeholder interactions, adaptation practices and other issues emerging from participants' accounts. Both descriptive and interpretive codes were used to capture participants' explicit accounts as well as the underlying meanings relevant to the study objective. Third, related codes were grouped into categories and potential themes.

Fourth, themes were reviewed and refined through constant comparison within and across transcripts to ensure coherence and consistency. The research team compared emerging patterns across IDIs and FGDs and considered similarities and differences in accounts across participant groups. Fifth, themes were defined and named to reflect their central organizing concepts. Finally, themes were organized into a coherent narrative that describes stakeholder perspectives on adaptive governance for climate change within the urban health system. The final themes were refined through iterative discussion among the research team and subsequently interpreted in relation to the Adaptive Governance Framework and the study objective.

### 2.5 Trustworthiness

Trustworthiness was established through strategies addressing credibility, transferability, dependability, and confirmability [37], [38], [39]. Credibility was strengthened through methodological triangulation using both IDIs and FGDs, as well as through member checking, in which selected participants reviewed preliminary findings and confirmed that the interpretations reflected their experiences and perspectives. Transferability was supported through detailed descriptions of the study setting, participant characteristics, and research procedures. Dependability was enhanced through transparent documentation of data collection, coding, theme development, and analytical decisions. Confirmability was strengthened by maintaining an audit trail comprising transcripts, field notes, coding records, and analytic memos, allowing findings to be traced directly to the data.

### 2.6 Research team and reflexivity

Data collection was conducted by all the authors; the principal investigator who isa female doctoral researcher with prior training and experience in qualitative research methods and health system governance, led the discussion or data collection process, then the second author was responsible for note-taking and logistical support during interviews and focus group discussions. Before data collection, the research team underwent refresher training on qualitative interviewing techniques, ethical conduct of research, and the management of climate-health-related discussions. No prior professional or personal relationships existed between the researchers and participants. Participants were informed about the study's objectives, the researchers' academic affiliations, and the purpose of the research before providing consent. Throughout the study, reflexive practices were employed to minimize potential researcher influence on data collection and interpretation as recommended [40]. Field notes and analytic memos were maintained to document observations, emerging reflections, and decisions made during the research process.

### 2.7 Ethical considerations

Ethical approval for the study was obtained from the National Institute for Medical Research (NIMR), Tanzania (Ref: NIMR/HQ/R.8a/Vol.IX/5102). Written informed consent was obtained from all participants before data collection. Participants were informed of the study objectives, their right to withdraw at any time without consequences, and measures taken to protect confidentiality and anonymity. No personal identifiers were included in transcripts, reports, or publications. Audio recordings, transcripts, and related documents were stored securely, and access was restricted to the research team.

## 3 Results

### 3.1 Characteristics of participants

A total of 68 participants were included in the study, comprising stakeholders from health facilities (Hospitals, health centres and dispensaries), municipal health management teams, and academia. Participants represented diverse demographic and professional backgrounds. There was a near-balanced gender distribution, with 36 male and 32 female participants. Most participants held undergraduate qualifications (*n* = 40), while seven had postgraduate qualifications and 21 had diploma or certificate-level training. Most (*n* = 54) of the participants worked at health facilities, while 11 were Municipal health management team members and 3 were university lecturers. The majority had worked in their current institution for three years or more (*n* = 56), and 43 participants reported more than three years of experience in their current position. Detailed participant characteristics are presented in Table 1.

**Table 1:** Demographic and professional characteristics of study participants (*N* = 68).

Characteristic	Category	n (%)
Sex	Male	36 (53)
	Female	32 (47)
Highest educational level	Postgraduate	7 (10)
	Undergraduate degree	40 (59)
	Diploma/Certificate	21 (31)
Participant category	Municipal Health Management Team members	11 (16)
	Health facility staff	54 (79)
	University lecturers	3 (5)
Duration working in current institution	≤2 years	12 (18)
	≥3 years	56 (82)
Experience in current position	≤3 years	25 (37)
	>3 years	43 (63)
Total		68 (100)

### 3.2 Thematic findings

Analysis of the interviews and focus group discussions identified three interrelated dimensions of adaptive governance within Dar es Salaam's urban health system. Three interrelated themes were identified: (1) multi-sectoral networks and coordination (2) Institutional perspectives on community engagement and knowledge integration, and (3) leadership, institutional learning and responsiveness in climate adaptation governance. Across these dimensions, participants described adaptive governance as a relational and evolving process shaped by interactions among health and non-health sectors, community actors, and institutional leaders. The accounts highlighted both existing governance mechanisms and perceived gaps in coordination, communication, community engagement, policy operationalization, and the translation of information into timely action. The themes and sub-themes are summarized in Table 2.

**Table 2:** Summary of thematic findings and illustrative quotations.

Broad theme	Sub-theme	Illustrative quote
1. Multi-sectoral networks and coordination	Climate change adaptation as a cross-sectoral governance process	“When we think about adaptation, we have to consider how decisions in one sector affect outcomes in another. For example, water management decisions affect health, and livestock health affects human health… How do we collaborate with the livestock sector? How do we collaborate with the water sector? Because water, in terms of both quality and quantity, can affect health.” *(IDI – Lecturer 2)*
	Governance networks and coordination mechanisms	“Coordination exists. Departments meet under the municipal director, and daily coordination takes place. However, given the breadth of climate change issues, it would be beneficial to have a dedicated unit to monitor the situation… Joint efforts and reporting mechanisms would strengthen accountability.” *(IDI – Municipal Research Coordinator)*
2. Institutional perspectives on community engagement and knowledge integration	Perceived role of community and institutional knowledge	“The community knows things we do not know. They know which areas flood first, which water sources are contaminated, where mosquitoes breed. They have lived there for years. If we do not ask them, we are working blind. But if we bring their knowledge together with our technical knowledge, we can develop solutions that actually work.” *(IDI – Municipal Health Officer)*
	Institutional perspectives on community awareness and engagement	“We do awareness campaigns, but they are often one-off. We go to a community, we talk about cholera prevention, we distribute materials, and then we leave. Six months later, the practices have reverted. Awareness must be continuous, not just during outbreaks. It must be part of the routine work.” *(IDI – Municipal Nutrition Officer)*
3. Leadership, learning and institutional responsiveness in climate adaptation governance	Leadership in policy interpretation and operationalization	“While policies include relevant components, they need to be further operationalized, especially at the primary healthcare level. A health worker at a dispensary should be able to link daily practice with climate change issues. That connection is still weak… What's needed is leadership that can translate policy intention into practical guidance, and promote learning.” *(IDI – Municipal Research Coordinator)*
	Leadership in coordination and learning processes	“We have started a system where facilities that have experienced floods share their lessons with other facilities. The leadership at the municipal level coordinates this exchange. It is not formal training, but it is very powerful because it is practical. People learn from their peers who have faced the same challenges.” *(IDI – Municipal Health Officer)*
	Leadership for adaptive responses	“After every flood or outbreak, we should sit together and ask: what worked, what did not work, what should we do differently next time? But we do not have that culture. We move from one crisis to the next without learning. Leadership should ensure that we reflect and improve.” *(IDI – Lecturer 1)*

#### 3.2.1 Multi-sectoral networks and coordination

Across interviews and focus group discussions, participants described climate change adaptation as a governance issue that extends beyond the health sector and requires interaction among multiple actors operating at different levels of the system. Climate-related health risks were viewed as interconnected with environmental, water, livestock, urban development sectors, and public health, with participants emphasizing that actions in one sector could influence health outcomes in another sector. This theme encompasses two interconnected sub-dimensions: the conceptualization of adaptation as an inherently cross-sectoral governance process, and the formal and informal coordination mechanisms through which such cross-sectoral action is or could be realized in practice.

##### 3.2.1.1 Climate change adaptation as a cross-sectoral governance process

Participants emphasized that climate-related health challenges cannot be addressed solely through health-sector interventions. Instead, adaptation was described as requiring collaboration among institutions or sectors responsible for environmental management, water resources, livestock services, local government administration, and public health. A recurring perspective was that climate-sensitive health risks arise from interactions among human populations, environmental conditions, and animal health systems. Participants therefore described adaptation governance as requiring integrated approaches that address these interconnected domains. Participants frequently described adaptation governance as dependent on intersectoral relationships. Water quality management, disease surveillance, environmental protection, and livestock health were presented as examples of areas where actions by one sector influence outcomes in another sector including health. The interconnectedness of sectors was articulated not only as a theoretical proposition but as a lived reality that influenced how stakeholders understood their work and its limitations. One stakeholder reflected on how different sectors influence each other's outcomes:

“*When we think about adaptation, we have to consider how decisions in one sector affect outcomes in another. For example, water management decisions affect health outcomes, and livestock health affects human health…. When we have such a system, it helps us understand how we can work together… How do we collaborate with the livestock sector? How do we collaborate with the water sector? Because water, in terms of both quality and quantity, can affect health*.” (IDI – Lecturer two).

This understanding of intersectoral interdependence was highlighted across multiple participant groups. Another participant emphasized the practical implications of this interconnectedness for service delivery:

“*The health sector alone cannot handle climate change impacts. When floods occur, we see cholera outbreaks, but the causes are in water and sanitation infrastructure, in urban planning, in waste management. So we must work with municipal engineers, with water authority, with environment department. The challenge is that each sector has its own priorities and budget cycles. Coordination becomes essential but also very difficult because no one sector owns the problem*.” (IDI – Municipal Research Coordinator).

A health worker similarly described how the lack of cross-sectoral action manifested at the local level:

“*We tell people to boil water, but the problem is the water source itself is contaminated because of poor drainage and waste. We can give health education, but if the municipal council does not fix the drainage, if the water authority does not treat the water properly, our efforts are limited. We need the other sectors to act, not just the health sector*.” (FGD – Medical Doctor).

The recognition of intersectoral interdependence also extended to understanding how climate-related risks cascade across domains. One participant explained:

“*When we talk about climate-sensitive diseases, we are talking about a system. A heat wave affects not just human health but also livestock, and when livestock are stressed, they produce less milk, which affects nutrition, especially for children. It is all connected. So, adaptation must be planned together, not in isolation*.” (IDI – Municipal health Officer).

##### 3.2.1.2 Governance networks and coordination mechanisms

Participants identified a range of formal and informal coordination arrangements linking government departments, academic institutions, communities, and development partners. Existing municipal coordination structures were described as providing opportunities for information exchange and joint planning across departments. These structures included routine departmental meetings, technical working groups, and ad hoc committees convened in response to specific events or initiatives. Participants described these mechanisms as valuable but often insufficient for the complexity and urgency of climate-related challenges.

At the municipal level, participants reported that coordination occurred through routine meetings convened under local government leadership. However, stakeholders also highlighted the complexity of managing climate-related issues through existing structures due to the broad range of sectors involved. One municipal participant explained:

“*Coordination exists. Departments meet under the municipal director, and daily coordination takes place. However, given the breadth of climate change issues, it would be beneficial to have a dedicated unit to monitor the situation…. with a coordinator overseeing activities across departments. Joint efforts and reporting mechanisms would strengthen accountability. We need structured platforms to discuss health challenges so that service delivery remains effective*.” (IDI – Municipal Research Coordinator).

Participants described coordination and communication as important elements of adaptation governance. They referred to existing mechanisms that bring together actors from different departments and sectors, while also discussing the need for clear channels for information exchange during routine activities and emergency situations. Stakeholders also reflected on situations in which multiple actors engaged communities independently, resulting in overlapping activities and repeated messaging. One interviewee had the following to say:

“*You might find both the Ministry of Water and the Ministry of Health visiting the same community, delivering similar messages separately*… *coordination is not just about having structures but about actively managing relationships and information sharing so that the efforts support each other on complementary, not being duplicative*.” (IDI – Lecturer one).

Another participant provided a concrete example of how the lack of coordination created confusion and inefficiency:

“*During the last floods, we had the Red Cross, the municipal council, and several partners all distributing chlorine and hygiene kits in the same areas. Some households received multiple kits while others received none. It was not because of bad intentions but because there was no single coordination point. We need one map, one plan, one channel of communication*.” (IDI – Municipal medical officer).

Participants also identified opportunities for strengthening coordination through formal agreements and joint planning processes. One participant suggested:

“*What we need is a memorandum of understanding between the health sector and the water authority, so that information sharing and joint planning become mandatory, not just voluntary. When coordination depends on personal relationships, it is fragile. We need institutional arrangements that make coordination part of the system*.” (IDI – Municipal Health Officer).

#### 3.2.2 Institutional perspectives on community engagement and knowledge integration

Communities were described as important actors within adaptation governance systems rather than passive recipients of interventions. The participants insisted on the relationships between health institutions and communities, the role of local knowledge in identifying climate-related risks, and the importance of maintaining communication channels between communities and health authorities.

##### 3.2.2.1 Perceived role of community and institutional knowledge

Participants emphasized the complementary roles of community members and health professionals in addressing climate-related health challenges. Health workers were viewed as possessing technical expertise on disease patterns and prevention, while community members were described as having contextual knowledge of local environmental conditions and vulnerabilities. Several participants highlighted the importance of governance arrangements capable of bringing these forms of knowledge together in decision-making processes. One participant demonstrated:

“*Health professionals understand disease patterns, and community members understand local problems*.” (FGD – Health facility in charge 1).

This concise statement captured a widely shared perspective. Another participant elaborated on what community knowledge entails and why it is essential:

“*The community knows things we do not know. They know which areas flood first, which water sources are contaminated, where mosquitoes breed. They have lived there for years. If we do not ask them, we are working blind. But if we bring their knowledge together with our technical knowledge, we can develop solutions that actually work*.” (IDI – Municipal Health Officer).

This integration of knowledge systems was viewed as essential for developing locally relevant and effective adaptation strategies. These institutional participants suggested that stronger connections between health leaders, community leaders, and local governance structures could facilitate information exchange and improve responsiveness to emerging climate-related challenges. One participant reflected:

*“What matters is not just that we have community health workers, but that there are community voices to be heard in decision-making. When communities can communicate their concerns and observations to health authorities, and when authorities respond to that information, that's when governance works.*” (IDI – Municipal Research Coordinator).

The importance of formalizing community participation structures was emphasized by another participant:

“*We do have village health committees, but often they are not fully utilized. They exist on paper, but they do not meet regularly, and their input is not always sought. We need to strengthen these structures so that community representatives are genuinely involved in planning and decision-making, not just informed after decisions are made*.” (IDI – Municipal Nutrition Officer).

Participants also highlighted the role of community health workers as knowledge brokers between communities and the formal health system:

“*Community health workers are the bridge. They live in the community, they know the families, they understand the local context. But they need support. They need training, they need supplies, they need supervision. If we invest in them, they can be the link that makes adaptation work at the community level*.” (IDI – Health Secretary 2).

##### 3.2.2.2 Institutional perspectives on community awareness within adaptation governance

Participants reflected on differences in community understanding of climate-related health risks. Limited awareness of environmental health practices was described as influencing how communities respond to climate-sensitive threats, particularly in densely populated and underserved settlements. These institutional stakeholders viewed public education and sustained engagement as important mechanisms for supporting community participation in adaptation activities:

*“Many squatter areas have people with low education levels. You find that having a toilet is not a priority for some people. Thus, increasing risk of water contamination during sustained rains.”* (FGD – Health facility nursing in charge).

Another participant described how limited awareness intersected with poverty and competing priorities:

“*When people are struggling to find food for the day, it is hard to think about environmental health. They know the water might not be safe, but they cannot afford to buy bottled water or to boil it if they do not have fuel. So they drink it anyway. Awareness is important, but we also need to address the underlying economic issues*.” (FGD – Health facility in charge 1).

Participants also observed that environmental conditions within communities – such as pollution from settlements, vehicles, and industries – can undermine prevention efforts when awareness is limited. This was described as reinforcing harmful practices unless sustained engagement is maintained:

“*Another challenge is limited community awareness. Environmental pollution from vehicles, industries, and settlements undermines prevention efforts. Without adequate community understanding, people revert to harmful practices. Addressing this requires strong collaboration and sustained awareness efforts*”. (IDI – Municipal Research Coordinator).

The challenge of sustaining awareness efforts over time was emphasized by another participant from the municipal health management team:

“*We do awareness campaigns, but they are often one-off. We go to a community, we talk about cholera prevention, we distribute materials, and then we leave. Six months later, the practices have reverted. Awareness must be continuous, not just during outbreaks. It must be part of the routine work*.” (IDI – Municipal Nutrition Officer).

Participants also reflected on the need for culturally appropriate and locally tailored awareness strategies:

“*You cannot use the same message for everyone. In some communities, people trust traditional healers more than health workers. In others, religious leaders are the most influential. We need to work with those trusted figures, not against them. Awareness must be delivered through channels that people trust*.” (FGD – Medical Doctor).

#### 3.2.3 Leadership, learning and institutional responsiveness in climate adaptation governance

Participants described leadership as an important component of adaptive governance, especially in facilitating coordination, interpreting policies, mobilizing resources, and responding to emerging climate-related risks. Their accounts focused on how governance institutions translate policy direction into operational actions and maintain responsiveness within changing environmental conditions. This theme encompasses three interconnected sub-dimensions: leadership in policy interpretation and operationalization, leadership in coordination and learning processes, and leadership for adaptive responses to climate-related risks.

##### 3.2.3.1 Leadership in policy interpretation and operationalization

Participants perceived difficulties in interpreting and operationalizing policies and guidelines, particularly among frontline health workers. Some reported inconsistencies in how similar policies are applied across institutions. A Municipal CHMT member noted that climate-related components within policies need to be translated into meaningful practice through leadership and learning processes:

“*While policies include relevant components, they need to be further operationalized, especially at the primary healthcare level. A health worker at a dispensary should be able to link daily practice with climate change issues. That connection is still weak…. What's needed is leadership that can translate policy intention into practical guidance, and promote learning so that healthcare workers can have the adaptative understanding and skills they need.* ” (IDI – Municipal Research Coordinator).

The gap between policy and practice was described as particularly acute at the frontline level. Another participant explained:

“*The national climate change strategy talks about health, but when you go to a dispensary, the health worker does not know what that means for their daily work. They have not been trained. They do not have guidelines. They are dealing with patients, not with policy documents. We need to bring the policy down to their level*.” (IDI – Municipal Health Secretary 1).

A frontline health worker described the challenges they faced in connecting policy to practice:

“*We hear about climate change on the radio, we attend meetings where it is mentioned, but no one has told us exactly what we should do differently in our work. Should we screen for different diseases? Should we change our health education messages? We need practical guidance, not just policy statements*.” (FGD – Health facility in charge 2).

The role of leadership in creating enabling conditions for policy implementation was emphasized:

“*Leadership at the facility level is crucial. If the in-charge understands climate change and its health implications, they can support their staff to integrate it into their work. But if the in-charge does not understand or does not prioritize it, nothing happens. So we need to build leadership capacity at all levels*.” (IDI – Municipal Research Coordinator).

##### 3.2.3.2 Leadership in coordination and learning process

Participants frequently referred to leadership as an important mechanism for coordinating actors, facilitating communication, and promoting adaptation responses across governance levels. Participants perceived leadership as involving the ability to facilitate learning and adaptation across governance levels. Rather than simply issuing directives, effective leaders were described as creating conditions for knowledge exchange, experimentation, and collective problem-solving. One participant explained:

“*When we say about leadership in this context is about helping people understand how their work connects to climate risks and adaptation. Not just about giving orders or distributing guidelines and manuals; it's about facilitating learning so that health workers at all levels can see how climate change affects their daily practice, and know what they can*.” (IDI – Municipal Research Coordinator).

Another participant had a similar observation emphasizing the need for clear coordination and communication structures:

“*Once we have such systems, they make it easier for us to understand the flow of information from the higher levels down to the final beneficiaries*.” (IDI – Lecturer one).

This perspective on leadership emphasized the importance of creating learning environments where frontline workers can develop competencies for addressing climate-related health challenges. The concept of learning was described as extending beyond formal training to include peer learning, reflective practice, and the documentation and sharing of experiences. One participant described how leadership could facilitate learning across facilities:

“*We have started a system where facilities that have experienced floods share their lessons with other facilities. The leadership at the municipal level coordinates this exchange. It is not formal training, but it is very powerful because it is practical. People learn from their peers who have faced the same challenges*.” (IDI – Municipal Health Officer).

Another participant emphasized the importance of creating safe spaces for experimentation and reflection:

“*If we want adaptation, we must allow people to try new things, to make mistakes, and to learn from them. Leadership must create that space. If people are afraid of being punished for failures, they will not innovate. They will just follow the old ways.*” (IDI – Lecturer two).

The role of leadership in bridging vertical and horizontal communication was also highlighted:

“*The municipal director has a key role. They can bring together the different departments, but they also can link us to the regional and national levels. When the municipal director prioritizes climate and health, it becomes a priority for everyone. Leadership matters*.” (IDI – Municipal Environmental Officer).

##### 3.2.3.3 Leadership for adaptive responses

Participants described leadership as essential for mobilizing coordinated responses to climate-related risks. Effective leadership was associated with establishing collaborative structures, strengthening preparedness mechanisms, and facilitating adaptation planning across sectors. Several participants highlighted the importance of multidisciplinary approaches to climate-related challenges, emphasizing the need for expertise from different professional backgrounds to support adaptation processes. Beyond simply establishing teams, participants emphasized that effective leadership involves creating processes for continuous learning and adaptation. One health facility participant noted:

“*It is not enough to have a multidisciplinary team. You need leadership that can facilitate ongoing learning so that team members learn from each other and from experience. This is how adaptive capacity develops through continuous reflection and adjustment*.” (FGD – Medical Doctor).

Participants also identified early warning systems as important governance instruments for anticipating and responding to climate-related hazards. However, they emphasized that the effectiveness of such systems depends on leadership processes that enable timely action. The gap between early warning information and actual response was a recurring concern. One participant observed that:

“*Developing early warning systems in our country. For events like heat waves, floods, and other disasters, robust early warning systems can minimize public health impacts*…. Developing early warning systems requires leadership that can coordinate across sectors, ensure that information flows to those who need it, and create processes for translating warnings into action. It's not just about having the systems; we should have leadership that makes them work.” (FGD – Medical Doctor)”.

Another participant described the challenges they faced in responding to early warnings:

“*We receive weather forecasts from the meteorology department, but they are not always translated into health risks. We are told it will rain heavily, but not what that means for cholera or for mosquito breeding. And even when we know, we do not always have the resources to act. We need not just information but also the capacity to respond*.” (IDI – Municipal Health Officer).

Participants also highlighted the need for after-action reviews and systematic learning from past events:

“*After every flood or outbreak, we should sit together and ask: what worked, what did not work, what should we do differently next time? But we do not have that culture. We move from one crisis to the next without learning. Leadership should ensure that we reflect and improve*.” (IDI – Lecturer one).

The role of leadership in mobilizing resources for adaptive responses was also emphasized:

“*When there is a cholera outbreak, the municipal director can release emergency funds. But for preparedness, for the things we need to do before the outbreak, it is harder to get resources. We need leadership that can advocate for resources for prevention, not just for response.*” (IDI – Municipal Health Officer).

A health facility in-charge described how leadership had facilitated a coordinated response during a recent flood event:

“*During the floods last year, the municipal director called an emergency meeting. All departments were there - health, water, planning, social welfare. We coordinated our response. It was not perfect, but it was better than before. The leadership made the difference. When the director takes it seriously, everyone takes it seriously*.” (FGD – Health facility in charge 1).

## 4 Discussion

This study explored stakeholder perspectives on adaptive governance for climate change adaptation within the urban health system of Dar es Salaam, Tanzania. Rather than viewing adaptive governance simply as the presence of coordination structures, participatory mechanisms, or leadership arrangements, the findings demonstrate that stakeholders perceive climate adaptation as a governance as a dynamic social process influenced by relationships, knowledge exchange, and learning, not only the presence of institutional structures or resources. Three interconnected governance dimensions emerged from the analysis: multi-sectoral networks and coordination; Institutional perspectives on community engagement and knowledge integration; and leadership, institutional learning and responsiveness in climate adaptation governance. Taken together, these findings suggest that the adaptive capacity of an urban health governance system depends not only on whether governance structures exist, but also on whether those structures are able to connect otherwise fragmented actors, facilitate learning across institutional boundaries, and convert knowledge into timely and coordinated action. These findings are consistent with the adaptive governance theory, which emphasizes networks, participation, flexibility, and learning as critical elements for managing complex social-ecological challenges such as climate change [16], [17]. However, our findings also highlight an important contextual dimension: the principles of adaptive governance may not operate automatically when they are introduced into hierarchical, sectoral fragmented, and resource-constrained health systems. Their practical value depends on the institutional relationships and leadership processes through which these principles are enacted.

A key finding of this study is the central role of multi-sectoral networks and coordination in climate adaptation governance. There is a consistent view of climate-related health risks as interconnected with environmental management, water systems, livestock health, urban planning, and local government functions. This reflects the growing recognition that climate-sensitive health challenges emerge from interactions across multiple sectors and therefore require governance arrangements that transcend traditional institutional boundaries [16]. Similar findings have been reported in studies of climate adaptation governance in the LMICs, where fragmented sectoral approaches have often limited effective responses to complex climate risks [2], [41], [42]. The findings, however, add an important qualification to this literature and to the Adaptive Governance Framework. The existence of coordination structures does not necessarily constitute adaptive governance. Participants described routine municipal meetings and other coordination mechanisms, but also identified overlapping activities, fragmented information flows, and the absence of sufficiently dedicated mechanisms for climate-related coordination. This distinction suggests that the adaptive value of networks lies less in their formal existence than in their capacity to facilitate meaningful relationships, information exchange, joint problem-solving, and coordinated action. From an adaptive governance perspective, such networks are important because they enable diverse institutions to mobilize knowledge, coordinate responses, and address uncertainty through collective action rather than isolated sectoral interventions [16], [17], [43], [44]. In Dar es Salaam, our findings suggest that this networking function is constrained by institutional boundaries and sector-specific responsibilities. Thus, adaptive governance appears to require not simply more coordination structures, but mechanisms that can bridge existing institutional fragmentation and create shared accountability for risks that do not fit within a single sectoral mandate.

This provides a contextual refinement of the network component of the Adaptive Governance Framework [17]. In a complex urban health environment, networks may be necessary but insufficient: their effectiveness depends on the ability of actors to move beyond parallel sectoral activities towards sustained information sharing, joint interpretation of risks, and coordinated decision-making. The implication is that adaptive governance should be understood as a quality of interaction among institutions, rather than only as the number or presence of intersectoral platforms [17].

Our findings also emphasize the importance of community engagement as a governance interface linking institutions with local populations. Institutional stakeholders in this study perceived community participation as an important component of adaptive governance. Communities were described as active contributors to adaptation processes through local knowledge, risk identification, and participation in prevention and response activities. This aligns with adaptive governance scholarship, which emphasizes the value of integrating multiple forms of knowledge and fostering participation across governance levels [16], [41]. The study further highlights the importance of linking technical expertise from health professionals with contextual knowledge held by community members. Participants perceived locally situated community knowledge as potentially complementary to technical health-sector knowledge. Such interactions can strengthen the responsiveness of adaptation strategies by ensuring that interventions are informed by local realities and emerging risks [2], [45]. In rapidly urbanizing settings characterized by informal settlements and diverse vulnerabilities, community engagement may therefore serve as a critical mechanism for enhancing the legitimacy, relevance, and effectiveness of climate adaptation efforts [45], [46], [47].

However, the findings require careful interpretation because community members were not included as participants in this study. The finding therefore does not demonstrate that communities themselves experience meaningful participation or that local knowledge is routinely incorporated into decision-making. Rather, it demonstrates that the health system and institutional stakeholders recognize community engagement and locally situated knowledge as potentially important components of adaptation governance. This distinction is theoretically important. Participation within an adaptive governance system may be formally recognized without necessarily resulting in meaningful influence over decisions. For example, a study of municipal managers in cities along Florida's east coast, found that although civil society actors may articulate climate-related concerns in public arenas, these perspectives do not necessarily reach or influence policy decision-making [48]. Thus, the existence or recognition of community engagement does not necessarily ensure that local knowledge and priorities influence formal adaptation decisions [48].

The findings therefore refine the participation component of the Adaptive Governance Framework by drawing attention to the difference between the recognition of community participation and the actual exercise of community influence [49], [50]. In the present context, community engagement appears to be mediated through health workers, local government structures, and other institutional actors. Such mediated participation may facilitate communication between communities and institutions, but it may also filter which forms of local knowledge reach decision-makers and how those concerns are interpreted. Direct engagement with communities is therefore important if adaptive governance is to move beyond institutional perceptions of participation towards more inclusive processes of knowledge production and decision-making.

This issue is particularly relevant in Dar es Salaam, where rapid urbanization, informal settlements, environmental inequalities, and differing levels of access to public services may produce highly localized experiences of climate-related health risks [7]. Governance mechanisms designed at municipal or sectoral levels may not fully capture these differences. Consequently, the adaptive governance principle of participation may require institutional mechanisms that allow locally experienced risks to enter formal planning and decision-making processes rather than relying only on top-down communication of adaptation messages.

Another important finding relates to the role of leadership in facilitating learning, coordination, and institutional responsiveness. The study has revealed leadership as essential for interpreting policies, promoting communication across governance levels, supporting multidisciplinary collaboration, and strengthening preparedness for climate-related hazards. These findings are consistent with adaptive governance scholarship, which identifies leadership as important for connecting actors, fostering collaboration, and supporting learning processes [9], [17], [44].

The findings extend this understanding by suggesting that, within the urban health-system context, leadership operates at the intersection of formal authority and relational work [51]. Participants did not describe effective leadership simply as issuing directives or enforcing policies. Instead, they emphasized leaders' roles in translating broad policy commitments into operational guidance, facilitating knowledge exchange, connecting different actors, and creating conditions for frontline learning. Leadership therefore appeared to function as a bridge between policy, institutions, knowledge, and action [16]. This is particularly relevant where climate change has not yet been fully translated into routine primary healthcare practice. The gap identified by participants between policy recognition and frontline operationalization suggests that adaptive governance can be constrained even when appropriate policies exist. Leadership becomes important because it can determine whether climate-related priorities are interpreted as additional policy requirements or integrated into everyday organizational learning and practice. In this sense, leadership does not only support adaptive governance; it helps connect the different elements through which adaptation becomes operational [52].

Across the three themes, several contextual barriers emerged that may constrain the functioning of adaptive governance in Dar es Salaam. First, institutional fragmentation may limit the ability of actors to respond to climate-related health risks that cross conventional sectoral boundaries. Water, environmental management, livestock, urban planning, and health are governed through different institutional mandates, which can create challenges for shared planning and accountability [16]. Second, information may not consistently move across governance levels in ways that support timely decision-making. Third, the translation of climate-related policy commitments into routine practice at lower levels of the health system appears to remain uneven.

These barriers are important because they demonstrate that adaptive governance is not simply a matter of adopting participatory or network-based arrangements. The ability to adapt depends on whether institutional structures allow actors to communicate across boundaries, whether leaders can facilitate learning, whether information can be converted into action, and whether affected populations have meaningful opportunities to influence decisions [48]. In a rapidly growing city such as Dar es Salaam, these conditions may be particularly important because climate-related risks intersect with existing urban inequalities, infrastructure pressures, and service-delivery constraints [7], [25].

The findings have important implications for strengthening climate-resilient urban health systems in Tanzania and other rapidly growing cities facing increasing climate-related health risks. While climate adaptation policies and sectoral initiatives continue to expand, the effectiveness of these efforts may depend on the extent to which governance systems can foster collaboration, participation, and institutional learning. The implications of the findings extend beyond establishing additional coordination platforms or strengthening individual institutional capacities. Efforts should also focus on improving the quality of relationships among institutions, creating mechanisms for shared interpretation of climate risks, strengthening feedback between governance levels, and supporting leaders to facilitate institutional learning.

At the system level, this could include strengthening cross-sectoral coordination mechanisms with clearly defined roles and accountability; establishing routine processes for sharing climate-related health information across sectors and governance levels; integrating climate considerations into routine primary healthcare planning and supervision; and developing mechanisms through which community-generated information can inform formal decision-making. Leadership development should similarly emphasize facilitation, learning, communication, and adaptive problem-solving alongside conventional administrative responsibilities. These implications are consistent with the Adaptive Governance Framework but also emphasize that the framework's principles require enabling institutional conditions to become operational. In resource-constrained urban health systems, adaptive governance may therefore be strengthened not only by creating new structures but by improving how existing structures learn, communicate, coordinate, and respond to emerging risks.

### 4.1 Strengths and limitations

This study has several strengths. First, it provides novel empirical evidence on climate change adaptation governance within an urban health system in SSA using the Adaptive Governance Framework, a perspective that has rarely been applied in studies of urban health systems in low- and middle-income countries. Second, the study included a diverse range of stakeholders, including municipal health managers, healthcare providers, and academic experts, allowing the capture of perspectives from multiple governance levels and institutional settings. Third, the use of both IDIs and FGDs enabled methodological triangulation and provided a comprehensive understanding of stakeholder perspectives on adaptation governance. The inclusion of participants from different municipalities and health facility levels further enhanced the breadth of experiences represented in the findings.

Several limitations should also be considered. First, although the study included perspectives from the health sector, and academia, representation of non-health sectors involved in climate adaptation governance was limited, with no direct representation from sectors such as water and livestock. This is particularly relevant to the findings on multi-sectoral coordination, which therefore primarily reflect the perspectives of health-sector and related institutional actors rather than a balanced account across all sectors involved in adaptation governance. Second, community members and local community leaders were not included as study participants. Consequently, findings concerning community engagement, community knowledge, and participation represent the perceptions and interpretations of institutional stakeholders rather than direct accounts from communities themselves. The study therefore cannot establish how community members understand, experience, or evaluate their participation in climate adaptation governance, nor independently verify the extent to which local knowledge is incorporated into decision-making. Future studies should deliberately include community representatives and other non-institutional actors, ideally using participatory approaches, to examine these processes from multiple perspectives.

Third, the findings may be subject to social desirability bias, particularly where participants described institutional coordination, leadership, and organizational responsiveness. Participants may have emphasized positive aspects of institutional practices or provided accounts that were considered socially or professionally acceptable. Fourth, because interviews and FGDs were conducted in Kiswahili and subsequently translated into English for analysis and reporting, some contextual or linguistic information may have been lost during translation. The research team sought to minimize this risk through careful translation and review of the translated transcripts. Finally, because the study was a secondary qualitative analysis of an existing dataset, the original sampling and data collection were not specifically designed to capture community members' perspectives on adaptive governance. Nevertheless, the findings provide useful insights into how institutional actors understand governance processes and relationships relevant to climate adaptation within an urban health system and identify areas for further investigation involving a broader range of sectors and actors.

### 4.2 Conclusion and recommendation

This study highlights the importance of adaptive governance in shaping climate change adaptation within urban health systems. Stakeholders in Dar es Salaam described climate adaptation as a process that depends on multi-sectoral coordination, community participation, and leadership-driven institutional responsiveness. The findings demonstrate that effective adaptation extends beyond sector-specific interventions and requires governance arrangements that facilitate collaboration, knowledge integration, learning, and collective action across multiple actors and governance levels. Guided by the Adaptive Governance Framework, the study contributes evidence on how stakeholders perceive the governance processes that support climate resilience in urban health systems. Strengthening adaptive governance mechanisms may enhance the ability of rapidly growing cities, particularly in developing countries to anticipate, respond to, and adapt to climate-related health risks while building more resilient and responsive health systems. By focusing on governance processes and stakeholder perceptions, this study complements existing research on institutional capacity gaps by explaining how governance functions through collaboration, participation, learning, and adaptation.

## Data sharing statement

The data are available from the corresponding author upon reasonable request.

## CRediT authorship contribution statement

**Lawrencia D. Mushi:** Writing – review & editing, Writing – original draft, Visualization, Validation, Supervision, Software, Resources, Project administration, Methodology, Investigation, Funding acquisition, Formal analysis, Data curation, Conceptualization. **Noel O. Ogweyo:** Writing – review & editing, Project administration, Methodology, Investigation, Data curation, Conceptualization.

## Declaration of competing interest

None declared.

## Appendix A Supplementary data

Supplementary material 1: FGD Guide.

Supplementary material 2: ICMJE Disclosure Form.

Supplementary material 3: IDI Guide 1 -Health system stakeholders.

Supplementary material 4: IDI Guide 2 -University stakeholders.
