# Supplementary material for: Adaptive governance for climate-resilient urban health systems: qualitative insights from stakeholders in Dar es Salaam, Tanzania
Source: Dialogues Health. 2026 Oct 5;9:100359. doi: 10.1016/j.dialog.2026.100359 (PMC13676699; doi:10.1016/j.dialog.2026.100359)
Supplement: Supplementary file 1 [file mmc1.docx]

## Section 1: Introduction and Ground Rules

Dear Participants,

Greetings

Thank you all for participating in this focus group discussion. We are conducting a study on the governance of climate change actions in urban health systems in Dar es Salaam. Your insights and experiences are essential for helping us understand how climate change affects your work, your communities, and the healthcare system. The discussion is expected to last approximately 60 to 90 minutes.

Your participation is voluntary, and all responses will remain confidential. There are no right or wrong answers; we are interested in your honest opinions. We kindly request that only one person speaks at a time and that everyone respects one another's views.

With your permission, we would like to record this session to enable precise note-taking. May we proceed?

**Preliminary information**

| Facility/group ID | Facility level and name | Council/district | Catchment area/population | Years of existence | Number of participants in the group |
| --- | --- | --- | --- | --- | --- |
|  |  |  |  |  |  |

## Section 2: FGD Discussion Themes and Questions

### 1. Local Experiences with Climate Impacts

Main Question:

- What changes in weather or climate patterns have you noticed in your community in recent years?

Probing Questions:

- How have these changes impacted people’s health?
- Are there specific health problems that have become more common?
- How do you believe climate change impacts your daily work in health service delivery or community health?

### 2. Preparedness and Response in Health Systems

Main Question:

- How do your health facilities or local authorities address climate-related health issues?

Probing Questions:

- Are there any emergency plans or protocols in place?
- Have you had any training or guidance on managing climate-related health events?
- How effective do you believe the response systems to be?

### 3. Awareness of Policies and Procedures

Main Question:

- Are you aware of any policies, plans, or guidelines that address climate change and its effects on health?

Probing Questions:

- Who developed these policies, and how are they communicated to health workers?
- Are the policies practical and straightforward to follow?
- Do you feel involved or informed in the policy process?

### 4. Stakeholder Collaboration and Governance

Main Question:

- Who are the key actors or institutions you collaborate with to tackle climate-related health issues?

Probing Questions:

- Do you collaborate with municipal officials, non-governmental organisations, or environmental agencies?
- How would you describe the level of coordination or support you receive?
- What challenges exist in working together with other sectors?

### 4. Community-Based Solutions and Local Knowledge

Main Question:

- Have there been any successful local initiatives to address the health impacts of climate change?

Probing Questions:

- What made these initiatives effective?
- Were they developed by the community or assisted by external actors?
- Can these be expanded or shared with other areas?

### 6. Suggestions for Improvement

Main Question:

- What do you believe ought to be done to enhance governance of the health system in addressing climate change?

Probing Questions:

- What role should community health workers play?
- What type of support or resources do you require?
- How can the health sector be better prepared for future climate events?

## Section 3: Closing

Thank you all for your participation. Your contributions are invaluable to this research. We appreciate your time and honesty. If you would like to receive a summary of the study results, please inform us after the session.

**Sehemu ya 1: Utangulizi na Kanuni za Msingi**

Ndugu washiriki

Tunawasalimu.
Asanteni sana kwa kushiriki katika mjadala huu wa kikundi. Tunafanya utafiti kuhusu utawala wa hatua dhidi ya mabadiliko ya tabianchi katika mifumo ya afya mijini, hususan katika jiji la Dar es Salaam. Uelewa na uzoefu wenu ni muhimu sana katika kutusaidia kufahamu jinsi mabadiliko ya tabianchi yanavyoathiri kazi zenu, jamii zenu, na mfumo wa utoaji huduma za afya.
Mjadala huu unatarajiwa kuchukua takribani dakika 60 hadi 90.
Ushiriki wenu ni wa hiari, na taarifa zote zitahifadhiwa kwa usiri mkubwa. Hakuna majibu sahihi au mabaya – tunachohitaji ni maoni yenu ya kweli. Tunawaomba kwa heshima kuwa mtu mmoja tu azungumze kwa wakati mmoja, na kila mmoja aheshimu maoni ya wengine.
Kwa ruhusa yenu, tungependa kurekodi kikao hiki ili kurahisisha uchukuaji wa kumbukumbu kwa usahihi. Je, tunaweza kuendelea?

**Taarifa za Awali**

| **Kitambulisho cha kituo/kikundi** | **Ngazi na jina la kituo** | **Halmashauri/Wilaya** | **Eneo la huduma/idadi ya watu** | **Miaka ya uwepo** | **Idadi ya washiriki kwenye kikundi** |
| --- | --- | --- | --- | --- | --- |
|  |  |  |  |  |  |

**Sehemu ya 2: Mijadala ya kwenye kundi na Maswali**

**1. Uzoefu wa Kijamii Kuhusu Athari za mabadiliko ya Tabianchi**

**Swali Kuu:**

- Ni mabadiliko gani ya hali ya hewa au tabianchi mmeyagundua katika jamii yenu katika miaka ya hivi karibuni?

**Maswali ya Kuchochea Majadiliano:**

- Mabadiliko haya yameathirije afya za watu?
- Kuna matatizo mahususi ya kiafya yaliyoanza kujitokeza mara kwa mara?
- Unadhani mabadiliko ya tabianchi yanaathirije kazi yako ya kila siku katika utoaji wa huduma za afya au afya ya jamii?

**2. Maandalizi na makabiliano katika Mifumo ya Afya ( dhidi ya adhari za mabadiliko ya tabia ya nchi)**

**Swali Kuu:**

- Vituo vyenu vya afya au mamlaka za mitaa hukabilianaje na matatizo ya kiafya yanayosababishwa na tabianchi?

**Maswali ya Kuchochea Majadiliano:**

- Je, kuna mipango ya dharura au taratibu maalum zilizowekwa?
- Umewahi kupatiwa mafunzo au mwongozo kuhusu kushughulikia matukio ya kiafya yanayohusiana na mabadiliko ya tabianchi?
- Je mfumo wa makabiliano yana ufanisi kiasi gani?

**3. Uelewa wa Sera na Taratibu**

**Swali Kuu:**

- Je, unafahamu kuhusu sera, mipango, au miongozo yoyote inayoshughulikia mabadiliko tabianchi na athari zake kwenye sekta ya afya?

**Maswali ya Kuchochea Majadiliano:**

- Nani waliandaa sera hizi, na zinawasilishwaje kwa watoa huduma za afya?
- Je, sera hizo ni rahisi kutekelezeka na kueleweka?
- Unajihisi umehusishwa au kupewa taarifa kwenye mchakato wa sera?

**4. Ushirikiano wa Wadau na Utawala**

**Swali Kuu:**

- Ni wadau au taasisi gani kuu mnazoshirikiana nazo kukabiliana na matatizo ya kiafya yanayosababishwa na tabianchi?
- Katika kituo chako cha kutolea huduma za afya, ni watu gani wanahusika na kusimamia wa maswala yahayohusiana na mabadiliko ya tabia ya nchi? Nini majukumu yao au wanafanyaje kazi?

**Maswali ya Kuchochea Majadiliano:**

- Je, mnashirikiana na maafisa wa manispaa, mashirika yasiyo ya kiserikali, au taasisi za mazingira?
- Unauelezeaje kiwango cha uratibu au msaada mnaopokea?
- Ni changamoto gani zinajitokeza katika kushirikiana na sekta nyingine?

**5. Suluhisho/mikakati za kijamii na uelewe wao**

**Swali Kuu:**

- Je, kumekuwa na juhudi au mikakati yoyote ya ndani ya jamii iliyofanikiwa kukabiliana na athari za kiafya za tabianchi?

**Maswali ya Kuchochea Majadiliano:**

- Ni mambo gani yaliyochangia mafanikio ya juhudi hizo?
- Je, zilianzishwa na jamii au ziliungwa mkono na wadau wa nje?
- Je, mikakati hiyo inaweza kupanuliwa au kushirikishwa maeneo mengine?

**6. Mapendekezo ya Uboreshaji**

**Swali Kuu:**

- Unadhani ni mambo gani yanapaswa kufanywa ili kuboresha utawala wa mfumo wa afya katika kukabiliana na mabadiliko ya tabianchi?

**Maswali ya Kuchochea Majadiliano:**

- Wafanyakazi wa afya ya jamii wanapaswa kuwa na nafasi gani?
- Ni aina gani ya msaada au rasilimali mnazohitaji?
- Sekta ya afya inaweza kujiandaa vipi vizuri zaidi kwa matukio ya tabianchi ya siku zijazo?

**Sehemu ya 3: Kufunga Mjadala**

Asanteni sana kwa kushiriki. Michango yenu ni ya thamani sana kwa utafiti huu. Tunathamini muda wenu na ukweli wa maoni yenu. Kama mnatamani kupata muhtasari wa matokeo ya utafiti huu, tafadhali tujulishe baada ya kikao hiki.
