# Supplementary material for: Adaptive governance for climate-resilient urban health systems: qualitative insights from stakeholders in Dar es Salaam, Tanzania
Source: Dialogues Health. 2026 Oct 5;9:100359. doi: 10.1016/j.dialog.2026.100359 (PMC13676699; doi:10.1016/j.dialog.2026.100359)
Supplement: Supplementary file 3 [file mmc3.docx]

## Section 1: Introduction and Informed Consent

Dear Participant

Thank you for agreeing to participate in this interview.

We are conducting a study on the governance of climate change actions in urban health systems, with a focus on Dar es Salaam. This interview aims to gain an understanding of your experiences, insights, and views on how climate adaptation is governed within your area of responsibility. Your participation is voluntary, and your responses will remain confidential.

May I proceed with the questions?

**Preliminary information**

| Interviewee’ ID | Gender | Role/position | Organization | Department/unit | Years of experience | Council/district |
| --- | --- | --- | --- | --- | --- | --- |
|  |  |  |  |  |  |  |

## Section 2: Interview Questions with Probes

### Part A: Understanding of Climate Change and Health Governance

1. In your own words, how do you understand climate change and its impact on urban health systems?

Probing Questions:

- How has climate change impacted public health outcomes or service delivery in your context?
- What specific health risks do you associate with climate change in urban areas like Dar es Salaam?
- How does your institution perceive the connection between climate and health?

### Awareness and Application of Policies

Main Question:

- Does your institution have any policies or frameworks in place to guide climate change adaptation in health?

Probing Questions:

- Which national or local policies are most relevant to your work?
- How are these policies applied at your institutional or operational level?
- Are there challenges in understanding or interpreting these policies?

### 1. Institutional Roles and Governance Structures

Main Question:

- Can you describe the role your institution plays in governing climate adaptation within the health sector?

Probing Questions:

- What are your department's specific responsibilities related to climate adaptation?
- How is decision-making around climate-health issues organized?
- Do you work together with other departments or agencies? How effective is this cooperation?

### 2. Implementation and Capacity Challenges

Main Question:

- What challenges do you encounter when implementing climate adaptation measures in your field of work?

Probing Questions:

- Are there challenges related to funding, staffing, or infrastructure?
- How would you assess your institution's ability to respond to climate-related health risks?
- Are there any regulatory or bureaucratic barriers that obstruct implementation?

### 3. Stakeholder Engagement and Coordination

Main Question:

- Who are the key stakeholders involved in your climate and health work, and how do you engage with them? What are their roles

Probing Questions:

- How would you describe your relationship with municipal authorities, NGOs, or the Ministry of Health?
- Are there coordination platforms or task forces where you gather to discuss climate and health strategies?
- What are the barriers to effective collaboration?

### 4. Best Practices and Opportunities

Main Question:

- Can you share any successful examples or best practices of climate adaptation in urban health systems?

Probing Questions:

- What made these initiatives successful in your view?
- Are there community-based or locally developed approaches that have been effective?
- Can these approaches be expanded or institutionalised?

### 5. Suggestions and Future Outlook

Main Question:

- What changes or improvements would you suggest to enhance the governance of climate change adaptation in urban health systems?

Probing Questions:

- What should policymakers or funders prioritize?
- What further support or capacity is required at the municipal or institutional level?
- How can your institution be better positioned to respond to future climate risks?

### Section 3: Closing

Thank you for your time and thoughtful responses. Your insights will be extremely helpful in understanding how governance can be enhanced to support climate adaptation in health systems. Would you be interested in receiving a summary of the findings once the study is complete?

**Sehemu ya 1: Utangulizi na Idhini ya Kujitolea**

**Ndugu Mshiriki,**
Asante kwa kukubali kushiriki katika mahojiano haya.
Tunafanya utafiti kuhusu utawala wa hatua dhidi ya mabadiliko ya tabianchi katika mifumo ya afya mijini, kwa kuzingatia jiji la Dar es Salaam. Lengo la mahojiano haya ni kupata uelewa wa uzoefu, maarifa, na mitazamo yako kuhusu jinsi mabadiliko ya tabianchi yanavyosimamiwa ndani ya eneo lako la uwajibikaji. Ushiriki wako ni wa hiari, na majibu yako yatadumishwa kwa usiri mkubwa.

**Je, naweza kuendelea na maswali?**

**Taarifa za Awali**

| **Kitambulisho cha Mhojiwa** | **Jinsia** | **Nafasi/Kazi** | **Taasisi** | **Idara/kitengo** | **Miaka ya Uzoefu** | **Halmashauri/Wilaya** |
| --- | --- | --- | --- | --- | --- | --- |
|  |  |  |  |  |  |  |

**Sehemu ya 2: Maswali ya Mahojiano na Maswali ya Kuchochea**

**Sehemu A: Uelewa wa Mabadiliko ya Tabianchi na Utawala wa Afya**

**1.** Kwa maneno yako mwenyewe, unaelewaje mabadiliko ya tabianchi na athari zake kwa mifumo ya afya mijini?

**Maswali ya Kuchochea:**

- Mabadiliko ya tabianchi yameathirije matokeo ya afya ya umma au utoaji huduma za afya katika muktadha/ mazingira yako?
- Ni hatari zipi mahsusi za kiafya unazozihusisha na mabadiliko ya tabianchi katika maeneo ya mijini kama Dar es Salaam?
- Taasisi yako inauelewaje uhusiano kati ya tabianchi na afya?

2. **Uelewa na Utekelezaji wa Sera**

**Swali Kuu:**

- Je, taasisi yako ina sera, kanuni, utaratibu au mifumo yoyote ya kuongoza mabadiliko ya tabianchi katika sekta ya afya?

**Maswali ya Kuchochea:**

- Ni sera zipi za nchi au za taasisi zinazohusiana zaidi na maswala ya mabadiliko ya tabia ya nchi?
- Sera hizo zinatekelezwaje katika ngazi ya taasisi au kituo cha kutolea huduma za afya?
- Kuna changamoto yoyote katika kupokea, kuelewa, kufasiri na kutekeleza sera hizo?

3. **Majukumu ya Taasisi na Miundo ya Utawala**

**Swali Kuu:**

- Je, unaweza kueleza jukumu la taasisi yako katika kusimamia mabadiliko ya tabianchi ndani ya sekta ya afya?

**Maswali ya Kuchochea:**

- Idara/idara yako inawajibika na mambo gani mahsusi yanayohusiana na mabadiliko ya tabianchi?
- Maamuzi kuhusu masuala ya tabianchi na afya yanatolewaje?
- Je, mnafanya kazi kwa pamoja na idara au mashirika mengine? Ushirikiano huo ni wenye mafanikio kiasi gani?

**3. Changamoto za Utekelezaji na Uwezo**

**Swali Kuu:**

- Ni changamoto zipi unazokutana nazo katika kutekeleza hatua za mabadiliko ya tabianchi katika kazi yako?

**Maswali ya Kuchochea:**

- Kuna changamoto zinazohusiana na ufadhili, rasilimali watu au miundombinu?
- Ungeitathminije uwezo wa taasisi yako kukabiliana na hatari za kiafya zinazotokana na tabianchi?
- Kuna vizuizi vya kisheria au kiutawala vinavyozuia utekelezaji?

**4. Ushirikiano wa Wadau na Uratibu**

**Swali Kuu:**

- Ni wadau gani wakuu wanaohusika katika kazi zako zinazohusiana na tabianchi na afya, na mnashirikianaje nao?

**Maswali ya Kuchochea:**

- Uhusiano wenu ukoje na mamlaka za miji, taasisi zisizo za kiserikali, au Wizara ya Afya?
- Je, kuna majukwaa ya uratibu au kamati kazi mnazokutana kujadili mikakati ya afya na tabianchi?
- Ni vikwazo gani vinazuia ushirikiano wenye ufanisi?

**5. Mifano ya Mafanikio na Fursa**

**Swali Kuu:**

- Je, ni ipi mifano yoyote ya mafanikio au mbinu bora za kukabiliana na mabadiliko ya tabianchi katika mifumo ya afya mijini?

**Maswali ya Kuchochea:**

- Nini kilichochangia mafanikio na jitihada hizo kwa mtazamo wako?
- Kuna mbinu zilizotokana na jamii au zilizoandaliwa na watu wa eneo husika ambazo zimekuwa na mafanikio?
- Je, mbinu hizi zinaweza kupanuliwa au kuingizwa rasmi katika mfumo?

**6. Mapendekezo na Mtazamo wa Baadaye**

**Swali Kuu:**

- Ni mabadiliko au maboresho gani unayopendekeza ili kuimarisha utawala wa kukabiliana na mabadiliko ya tabianchi katika mifumo ya afya mijini?

**Maswali ya Kuchochea:**

- Wapanga sera au wafadhili wanapaswa kupewa kipaumbele gani?
- Ni msaada au uwezo gani wa ziada unahitajika katika ngazi ya manispaa au taasisi?
- Taasisi yako inaweza kujiandaa vipi vyema zaidi kukabiliana na hatari za tabianchi zijazo?

**Sehemu ya 3: Hitimisho**

Asante kwa muda wako na majibu yako ya kina. Maarifa yako yatasaidia sana katika kuelewa jinsi utawala unaweza kuboreshwa ili kusaidia juhudi za kukabiliana na tabianchi katika mifumo ya afya.

**Jina la Hojaji………………………………………. Tarehe………………………………….**
