# Supplementary material for: Adaptive governance for climate-resilient urban health systems: qualitative insights from stakeholders in Dar es Salaam, Tanzania
Source: Dialogues Health. 2026 Oct 5;9:100359. doi: 10.1016/j.dialog.2026.100359 (PMC13676699; doi:10.1016/j.dialog.2026.100359)
Supplement: Supplementary file 4 [file mmc4.docx]

## Section 1: Introduction and Informed Consent

Dear Participant

Thank you for agreeing to participate in this interview.

We are conducting a study on the governance of climate change actions in urban health systems, with a focus on Dar es Salaam. This interview aims to gain an understanding of your experiences, insights, and views on how climate adaptation is governed within urban health system. Your participation is voluntary, and your responses will remain confidential.

May I proceed with the questions?

**Preliminary information**

| Interviewee’ ID | Gender | Role/position | Organization | Department/unit | Years of experience | Council/district |
| --- | --- | --- | --- | --- | --- | --- |
|  |  |  |  |  |  |  |

**Understanding of Climate Change and Health Governance**

1. In your own words, how do you understand climate change and its effects on urban health systems?
   **Probing questions:**
   • How has climate change affected public health outcomes or the delivery of health services in your context?
   • What specific health risks do you associate with climate change in urban areas such as Dar es Salaam?
   • How do you perceive the relationship between climate change and health systems?

**Awareness and Implementation of Policies**
**Main question:**
2. Do health systems have any policies or frameworks that guide climate change adaptation?
**Probing questions:**
• Which national or local policies are most relevant in addressing climate change within health systems, especially in urban areas?
• How are these policies applied at the institutional or operational level?
• Are there challenges in the formulation, understanding, interpretation, or implementation of these policies?

**Institutional Roles and Governance Structures**
**Main question:**
Can you describe the key actors and their roles in managing climate change adaptation within the health sector?
**Probing questions:**
• Which specific departments or units are responsible for addressing climate change issues in urban health systems?
• How are decisions on climate and health issues made?

**Implementation and Capacity Challenges**
**Main question:**
• What challenges do actors face when implementing climate change adaptation measures in urban health systems?
**Probing questions:**
• Are there challenges related to funding, staffing, or infrastructure?
• How would you assess the institution’s capacity to address health risks arising from climate change?
• Are there legal or administrative barriers that hinder implementation?

**Stakeholder Engagement and Coordination**
**Main question:**
Who are the key stakeholders involved in activities related to climate change in urban health systems? What roles do they play?
**Probing questions:**
• How would you describe the relationship between your institution and universities, municipal authorities, NGOs, or the Ministry of Health in governing climate change?
• Are there coordination platforms or special committees where universities meet to discuss climate and health strategies?
• What are the barriers to effective collaboration?

**Best Practices and Opportunities**
**Main question:**
Can you share any examples of success or best practices in climate change adaptation within urban health systems?
**Probing questions:**
• In your view, what contributed to the success of these efforts?
• Are there community-based or indigenous approaches that have proven effective?
• Can these approaches be expanded or institutionalized?

**Recommendations and Future Outlook**
**Main question:**
• What changes or improvements would you suggest to strengthen the governance of climate change adaptation in urban health systems?
**Probing questions:**
• What should policymakers or funders prioritize?
• What additional support or capacity is needed at the municipal or institutional level?
• How can your institution be better positioned to address future health risks arising from climate change?

### Section 3: Closing

Thank you for your time and thoughtful responses. Your insights will be extremely helpful in understanding how governance can be enhanced to support climate adaptation in health systems. Would you be interested in receiving a summary of the findings once the study is complete?

**Sehemu ya 1: Utangulizi na Idhini ya Kujitolea**

**Ndugu Mshiriki,**
Asante kwa kukubali kushiriki katika mahojiano haya.
Tunafanya utafiti kuhusu utawala wa hatua dhidi ya mabadiliko ya tabianchi katika mifumo ya afya mijini, kwa kuzingatia jiji la Dar es Salaam. Lengo la mahojiano haya ni kupata uelewa wa uzoefu, maarifa, na mitazamo yako kuhusu jinsi mabadiliko ya tabianchi yanavyosimamiwa ndani ya eneo lako la uwajibikaji. Ushiriki wako ni wa hiari, na majibu yako yatadumishwa kwa usiri mkubwa.

**Je, naweza kuendelea na maswali?**

**Taarifa za Awali**

| **Kitambulisho cha Mhojiwa** | **Jinsia** | **Nafasi/Kazi** | **Taasisi** | **Idara/kitengo** | **Miaka ya Uzoefu** | **Halmashauri/Wilaya** |
| --- | --- | --- | --- | --- | --- | --- |
|  |  |  |  |  |  |  |

**Sehemu ya 2: Maswali ya Mahojiano na Maswali ya Kuchochea**

Uelewa wa Mabadiliko ya Tabianchi na Utawala wa Afya

### 1. Kwa maneno yako mwenyewe, unaelewaje mabadiliko ya tabianchi na athari zake katika mifumo ya afya mijini?

### Maswali ya kuchochea majibu:

### Mabadiliko ya tabianchi yameathiri vipi matokeo ya afya ya umma au utoaji wa huduma za afya katika muktadha wako?

### Ni hatari zipi mahsusi za kiafya unazohusisha na mabadiliko ya tabianchi katika maeneo ya mijini kama Dar es Salaam?

### Unaonaje uhusiano kati ya hali ya hewa na afya?

### Uelewa na Utekelezaji wa Sera

### Swali Kuu:

### 2. Je, mifumo ya afya ina sera au mifumo yoyote inayoongoza katika kukabiliana na mabadiliko ya tabianchi kwenye afya?

### Maswali ya kuchochea majibu:

### Ni sera zipi za kitaifa au za kimitaa zinazohusiana zaidi katika kukabiliana na mabadiliko ya tabianchi kwenye mifumo ya afya, hasa ya mijini?

### Sera hizi zinatumikaje katika ngazi ya taasisi au utekelezaji?

### Je, kuna changamoto katika maandalizi, uelewa, tafsiri, au utekelezaji wa sera hizi?

### Majukumu ya Taasisi na Miundo ya Utawala

### Swali Kuu:

### Unaweza kuelezea wahusika wakuu na majukumu yao katika kusimamia hatua za kukabiliana na mabadiliko ya tabianchi ndani ya sekta ya afya?

### Maswali ya kuchochea majibu:

### Ni idara au vitengo vipi maalum vinavyohusika na masuala ya kukabiliana na mabadiliko ya tabianchi katika mifumo ya afya mijini?

### Maamuzi kuhusu masuala ya hali ya hewa na afya yanafanywaje?

### Utekelezaji na Changamoto za Uwezo

### Swali Kuu:

### Ni changamoto zipi wahusika wanakutana nazo wanapotekeleza hatua za kukabiliana na mabadiliko ya tabianchi katika mifumo ya afya mijini?

### Maswali ya kuchochea majibu:

### Je, kuna changamoto zinazohusiana na ufadhili, watumishi, au miundombinu?

### Unawezaje kutathmini uwezo wa taasisi kushughulikia hatari za kiafya zinazotokana na mabadiliko ya tabianchi?

### Je, kuna vikwazo vya kisheria au kiutawala vinavyokwamisha utekelezaji?

### 4. Ushirikiano na Uratibu wa Wadau

### Swali Kuu:

### Ni wadau gani wakuu wanaohusika katika kazi zinazohusiana na hali ya hewa na afya, na mnashirikianaje nao? Ni majukumu gani wanayotekeleza?

### Maswali ya kuchochea majibu:

### Unaelezeaje uhusiano kati ya taasisi yako na vyuo vikuu, mamlaka za manispaa, mashirika yasiyo ya kiserikali (NGOs), au Wizara ya Afya katika kusimamia mabadiliko ya tabianchi?

### Je, kuna majukwaa ya uratibu au kamati maalum ambapo vyuo vikuu hukutana kujadili mikakati ya hali ya hewa na afya?

### Ni vikwazo gani vinavyokwamisha ushirikiano wenye ufanisi?

### 5. Mifano Bora na Fursa

### Swali Kuu:

### Unaweza kushiriki mifano yoyote ya mafanikio au mbinu bora za kukabiliana na mabadiliko ya tabianchi katika mifumo ya afya mijini?

### Maswali ya kuchochea majibu:

### Kwa mtazamo wako, nini kilipelekea mafanikio ya juhudi hizo?

### Je, kuna mbinu za kijamii au za kienyeji ambazo zimeonyesha mafanikio?

### Je, mbinu hizi zinaweza kupanuliwa au kufanywa kuwa sehemu rasmi ya mifumo ya taasisi?

### 6. Mapendekezo na Mtazamo wa Baadaye

### Swali Kuu:

### Ni mabadiliko au maboresho gani ungependekeza ili kuimarisha utawala wa hatua za kukabiliana na mabadiliko ya tabianchi katika mifumo ya afya mijini?

### Maswali ya kuchochea majibu:

### Watunga sera au wafadhili wanapaswa kuweka kipaumbele kwyenye nini?

### Ni msaada au uwezo gani zaidi unahitajika katika ngazi ya jiji, manispaa au taasisi za afya?

### Ni kwa jinsi gani taasisi yako inaweza kujiweka vizuri zaidi kukabiliana na hatari za kiafya zitokanazo na mabadiliko ya tabianchi katika siku zijazo

**Sehemu ya 3: Hitimisho**

Asante kwa muda wako na majibu yako ya kina. Maarifa yako yatasaidia sana katika kuelewa jinsi utawala unaweza kuboreshwa ili kusaidia juhudi za kukabiliana na tabianchi katika mifumo ya afya.

**Jina la Hojaji………………………………………. Tarehe………………………………….**
